# Efficacy and safety of risankizumab versus methotrexate in patients with moderate-to-severe plaque psoriasis: results from IMMbrace, a randomized, double-blind, phase 3 study with an open-label extension period in Brazil^☆^

**DOI:** 10.1016/j.abd.2024.08.002

**Published:** 2024-12-07

**Authors:** Tania F. Cestari, Cacilda da Silva Souza, Luna Azulay-Abulafia, Ricardo Romiti, André V.E. Carvalho, Caio César Silva de Castro, Sílvio Alencar Marques, João Roberto Antonio, Lincoln Fabrício, Ahmed M. Soliman, Tianshuang Wu, Ranjeeta Sinvhal, Vassilis Stakias, Alexandra P. Song, Jasmina Kalabic, Naomi Martin, Luiza Keiko Matsuka Oyafuso

**Affiliations:** a Department of Dermatology, Hospital de Clínicas de Porto Alegre, Universidade Federal do Rio Grande do Sul, Porto Alegre, RS, Brazil; b Division of Dermatology, Faculdade de Medicina de Ribeirão Preto, Universidade de São Paulo, Ribeirão Preto, SP, Brazil; c Instituto de Dermatologia e Estética do Rio de Janeiro, Rio de Janeiro, Brazil; d Department of Dermatology, Hospital das Clínicas, Universidade de São Paulo, São Paulo, SP, Brazil; e Department of Dermatology, Hospital Moinhos de Vento, Porto Alegre, RS, Brazil; f Discipline of Dermatology, Pontifícia Universidade Católica do Paraná, Curitiba, PR, Brazil; g Department of Infectology, Dermatology, Imaging Diagnosis and Radiotherapy, Faculty of Medicine, Universidade Estadual Paulista, Botucatu, SP, Brazil; h Discipline of Dermatology, Faculdade Estadual de Medicina de São José do Rio Preto, São José do Rio Preto, SP, Brazil; i Dermaology Service, Hospital Universitário Evangélico Mackenzie de Curitiba, Curitiba, PR, Brazil; j AbbVie Inc., North Chicago, Illinois, United States; k AbbVie Deutschland GmbH & Co. KG, Ludwigshafen, Germany; l In memoriam; m Department of Dermatology, Faculdade de Medicina de ABC, São Paulo, SP, Brazil

**Keywords:** Methotrexate, Psoriasis, Risankizumab

## Abstract

**Background:**

Psoriasis, a chronic, inflammatory skin disease, requires long-term therapy. Risankizumab is a humanized immunoglobulin G1 monoclonal antibody that specifically inhibits interleukin 23 by binding to its p19 subunit.

**Objective:**

The authors assessed the efficacy and safety of risankizumab compared with methotrexate in adults with moderate-to-severe plaque psoriasis.

**Methods:**

IMMbrace was a phase 3, multicenter, randomized, double-blind, double-dummy, active-controlled study. Patients received subcutaneous risankizumab 150 mg at weeks 0, 4, and 16 plus oral placebo weekly, or oral methotrexate 5 mg weekly (with dose escalation up to 25 mg based on response and tolerability) plus subcutaneous placebo at weeks 0, 4, and 16. Primary efficacy endpoints were the proportions of patients who achieved ≥ 90% improvement in Psoriasis Area and Severity Index (PASI90) and static Physician’s Global Assessment of clear/almost clear (sPGA 0/1) at week 28. Safety was also assessed.

**Results:**

Among 98 patients randomized (risankizumab, n = 50; methotrexate, n = 48), 95 completed the double-blind period. At week 28, significantly higher proportions of patients treated with risankizumab versus methotrexate achieved PASI90 (84.0% vs. 35.4%; p < 0.001); sPGA 0/1 was achieved by 90.0% and 64.6% of patients in the risankizumab and methotrexate groups (p ≤ 0.001). Risankizumab efficacy was maintained throughout week 112. Adverse event rates were similar in the two groups.

**Study limitations:**

The sample size was small due to the difficulty of recruiting patients without methotrexate use.

**Conclusions:**

Risankizumab demonstrated superior efficacy over methotrexate at week 28; efficacy was maintained, and no new safety findings were observed through week 112.

## Introduction

Psoriasis is a chronic, immune-mediated inflammatory skin disorder causing red, scaly plaques and is associated with reduced quality of life.^1^, ^2^ The estimated prevalence in Brazil ranges from 1.1%–1.5%, with the highest indicators in the South and Southeast regions.^3^ The management of moderate-to-severe psoriasis includes controlling the disease activity while minimizing treatment-related side effects. Methotrexate has been widely used as a systemic therapy and has been the standard of care for psoriasis management in Brazil for decades; however, methotrexate is associated with various complications, including gastrointestinal, hepatic, renal, and hematopoietic abnormalities.^4^, ^5^, ^6^ A growing understanding of the disease pathogenesis has led to newer, target-specific biologic agents, including interleukin 23 antagonists.^7^

Risankizumab, a humanized immunoglobulin G1 monoclonal antibody that binds to the p19 subunit to specifically inhibit interleukin 23,^8^ has demonstrated superior clinical efficacy compared with placebo at week 16^9^, ^10^, ^11^ and against other psoriasis treatments, including ustekinumab,^10^ adalimumab,^12^ and secukinumab.^13^ Risankizumab is approved in Brazil (September 2020) and many other countries to treat adult patients with moderate-to-severe plaque psoriasis.^14^ However, to date, no clinical studies have compared risankizumab with traditional systemic agents such as methotrexate. Here, the authors report results from the first phase 3b head-to-head trial comparing the clinical safety and efficacy of risankizumab with methotrexate in adults with moderate-to-severe plaque psoriasis following the approval of risankizumab in Brazil.

## Methods

### Patients

Eligible patients were adults aged ≥ 18 years with a ≥ 6-month history of moderate-to-severe plaque psoriasis, defined as ≥ 10% body surface area involvement; Psoriasis Activity and Severity Index (PASI) of ≥ 12; and static Physician Global Assessment (sPGA) score of ≥ 3. Patients were candidates for treatment with methotrexate according to local labeling and were without clinically significant findings on chest X-rays. Patients were ineligible to enroll if they had non-plaque forms of psoriasis (including guttate, erythrodermic, or pustular); drug-induced psoriasis; evidence of chronic or relevant acute infections including HIV, viral hepatitis, and/or tuberculosis; an active or suspected malignancy or history of malignancy within 5 years before screening; any other relevant medical conditions (e.g., chronic alcohol or drug abuse, organ transplant, abnormal clinical laboratory values, history of hypersensitivity to a systemically administered biologic therapy); or active ongoing inflammatory diseases that might confound study evaluations according to the investigator’s judgment. Women of childbearing potential and all men were required to use contraception during the study and for at least 20 weeks (for women) or 3 months (for men) after the last dose of methotrexate/methotrexate placebo.

### Study design and treatment

**Figure 1:**
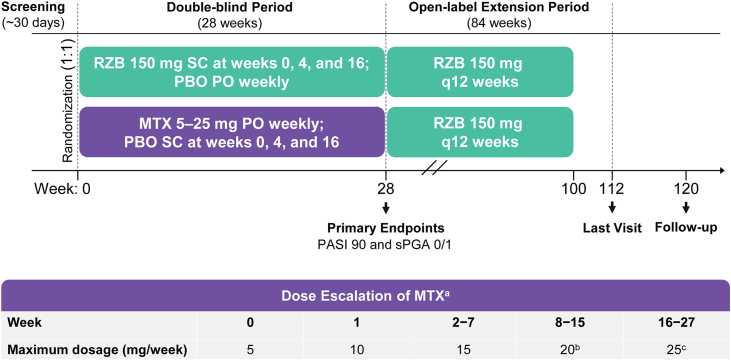
Study design. MTX, methotrexate; PASI90; ≥90% improvement in Psoriasis Area and Severity Index; PBO, Placebo; PO, by mouth; q12 weeks, every 12-weeks; RZB, risankizumab; SC, subcutaneous; sPGA 0/1, static Physician’s Global Assessment of clear or almost clear. ^a^ Oral MTX doses were also adjusted due to patient-reported symptoms, findings at physical examination, reported adverse events, and/or changes in clinical laboratory profiles, as deemed appropriate by the study investigators. ^b^ Starting at week 8, patients who did not achieve PASI90 or Spga 0/1 had their MTX dose increased by 5 mg (up to 20 mg per week) through week 15. ^c^ Starting at week 16, patients who did not achieve PASI90 or sPGA 0/1 had their MTX dose increased by 5 mg (up to 25 g per week) through week 27.

The IMMbrace study (ClinicalTrials.gov; NCT03219437) was a phase 3b, multicenter, randomized, double-blind, double-dummy, active-controlled clinical trial that compared the efficacy and safety of subcutaneous risankizumab with oral methotrexate in patients with moderate-to-severe plaque psoriasis. The study was conducted at 11 sites located in Brazil. The study design included a 30-day screening period, a 28-week double-blind treatment period, and an 84-week open-label treatment period (Fig. 1). In the double-blind period, patients were randomized 1:1 into two treatment groups to receive (in a double-dummy design) either Risankizumab 150 mg as subcutaneous injections at weeks 0, 4, and 16 plus placebo capsules by mouth weekly, or methotrexate capsules by mouth once weekly plus placebo injections at weeks 0, 4, and 16 (Fig. 1). Methotrexate dosing started at 5 mg and could be increased to 25 mg based on clinical response and tolerability. Investigators determined all methotrexate dosage adjustments (withholding, reducing, or increasing) based on patient-reported symptoms, findings on physical examination, adverse events (AEs), and/or changes in clinical laboratory profiles. From week 28, patients continuing in the study received open-label Risankizumab 150 mg every 12 weeks through week 100 (Open-Label Extension [OLE] period). The last study visit was at week 112; a follow-up telephone call occurred 20 weeks after the final subcutaneous injection or 4 weeks after the final oral administration, whichever was later.

The study was conducted in accordance with Good Clinical Practice guidelines as defined by the International Council for Harmonisation of Technical Requirements for Pharmaceuticals for Human Use guidelines, the Declaration of Helsinki, and all applicable guidelines and regulations governing clinical study conduct; all patients provided written informed consent. An independent ethics committee/institutional review board ensured the study's ethical, scientific, and medical appropriateness before the study was conducted and approved all relevant documentation.

### Assessments

#### Efficacy

The ranked primary efficacy endpoints were achievement of ≥ 90% improvement from baseline in PASI (PASI90) and sPGA of clear or almost clear (sPGA 0/1) at week 28. The ranked secondary efficacy endpoints (all assessed at week 28) were achievement of 100% improvement from baseline in PASI (PASI100), sPGA of clear (sPGA0), ≥ 75% improvement from baseline in PASI (PASI75), change from baseline in European Quality of Life 5 Dimensions (EQ-5D-5L) index score, and achievement of a ≥ 0.1-point increase (Minimal Clinically Important Difference [MCID]) from baseline in EQ-5D-5L index score. Using post hoc analyses, the authors evaluated change from baseline in EQ-5D-5L index score and achievement of MCID from baseline in EQ-5D-5L index score among patients with a baseline EQ-5D-5L index score ≤ 0.9 at week 28. An additional endpoint was the proportion of patients achieving a Dermatology Life Quality Index score of 0 (DLQI 0 [no effect on patient’s life]) at week 28.

#### Safety

Safety evaluations, including monitoring Treatment-Emergent AEs (TEAEs), analyzing results from standard laboratory tests, performing physical examinations, and conducting vital sign measurements, were performed throughout the study and findings were tabulated using Medical Dictionary for Regulatory Activities system organ class and preferred terms (version 24.0).

### Statistical analysis

A sample size of 100 patients was needed to achieve more than 95% power to detect the difference of 40% between risankizumab and methotrexate. Data were analyzed using SAS statistical software version 9.4 (SAS Institute Inc., Cary, NC, USA). All efficacy analyses were performed in the intent-to-treat population, which included all randomized patients. The ranked primary and secondary efficacy endpoints were analyzed in a hierarchical order using two-sided tests with a type 1 error of 0.05. Categorical endpoints were analyzed using the Cochran-Mantel-Haenszel test stratified by the study center; missing data were imputed using non-responder imputation for the primary analysis and non-responder imputation incorporating multiple imputations to handle missing data due to COVID-19 for the long-term analysis. Continuous endpoints were analyzed using a one-way analysis of covariance with treatment, study site, and baseline value in the model; the last-observation-carried-forward method was used to impute missing data. Safety was analyzed in all patients randomized at baseline who received at least one dose of the study drug.

## Results

### Patients

**Figure 2:**
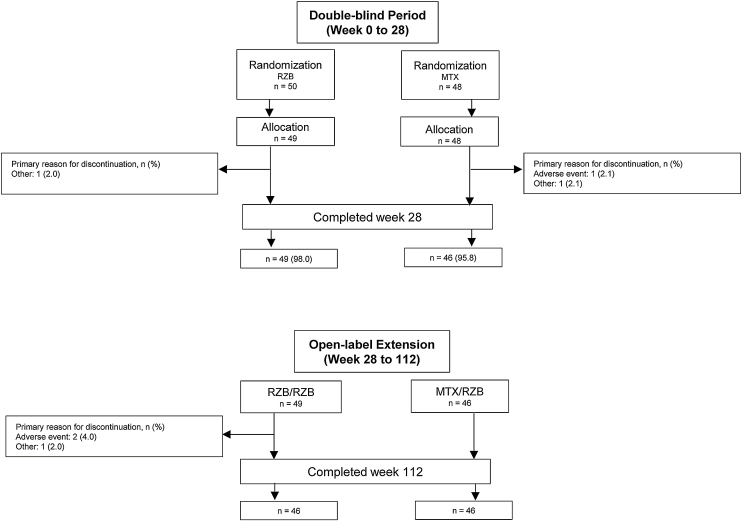
Patient disposition. MTX, Methotrexate; RZB, Risankizumab.

This study was conducted from July 2018 through November 2021. A total of 11 sites enrolled 104 patients in this study; however, one study site was closed for non-compliance with the protocol, and all patients enrolled at this site were excluded from efficacy and safety analyses. Of 98 patients randomized to receive risankizumab (n = 50) or methotrexate (n = 48) at compliant sites, 95 (96.9%) completed the double-blind period, and 92 (95.8%) completed the OLE period (Fig. 2). Six patients discontinued the study, including four in the risankizumab group (two due to an AE and two due to other reasons) and two in the methotrexate group (one due to an AE and one due to other reasons). Demographic and baseline disease characteristics were generally similar across treatment groups (Table 1). The mean (SD) patient age was 48.2 (14.4) years and 68.4% of patients were male. At baseline, patients had a mean PASI of 21.0 and a mean EQ-5D-5L index score of 0.8%; 83.7% and 16.3% of patients had moderate and severe sPGA scores, respectively.

**Table 1:** Baseline demographics and characteristics.

Parameter	RZB 150 mg (n = 50)	MTX 5–25 mg (n = 48)
Age, years, mean (SD)	48.3 (14.6)	48.2 (14.4)
Male, n (%)	35 (70.0)	32 (66.7)
Race, n (%)		
White	46 (92.0)	38 (79.2)
Black	3 (6.0)	9 (18.8)
Multiple^a^	1 (2.0)	1 (2.1)
Hispanic or Latino, n (%)	46 (92.0)	45 (93.8)
BMI, kg/m^2^, n (%)		
< 25	6 (12.0)	7 (14.6)
25 to < 30	19 (38.0)	17 (35.4)
≥ 30	25 (50.0)	24 (50.0)
Duration of plaque psoriasis, years, mean (SD)	16.6 (13.2)	12.4 (10.5)
BSA affected by psoriasis, mean (SD)	30.2 (16.9)	34.3 (17.5)
Median (range)	25.0 (10.0–70.0)	29.5 (10.0–80.0)
PASI, mean (SD)	20.0 (7.9)	22.1 (7.4)
Median (range)	15.3 (12.1–41.4)	19.4 (13.5–46.0)
sPGA, n (%)		
Moderate (score of 3)	43 (86.0)	39 (81.3)
Severe (score of 4)	7 (14.0)	9 (18.8)
EQ-5D-5L index score, mean (SD)	0.8 (0.2)	0.7 (0.3)
Median (range)	0.9 (0.1–1.0)	0.9 (-0.2–1.0)
DLQI, mean (SD)	12.3 (7.0)	13.1 (7.3)
Median (range)	12.0 (0–27.0)	13.5 (0–29.0)

BMI, Body Mass Index; BSA, Body Surface Area; DLQI, Dermatology Life Quality Index; EQ-5D-5L, European Quality of Life 5 Dimensions; MTX, Methotrexate; PASI, Psoriasis Area and Severity Index; RZB, Risankizumab; sPGA, Static Physician’s Global Assessment.

a Any patients identifying as more than one race were categorized as multiple.

### Efficacy

#### Primary analysis

**Figure 3:**
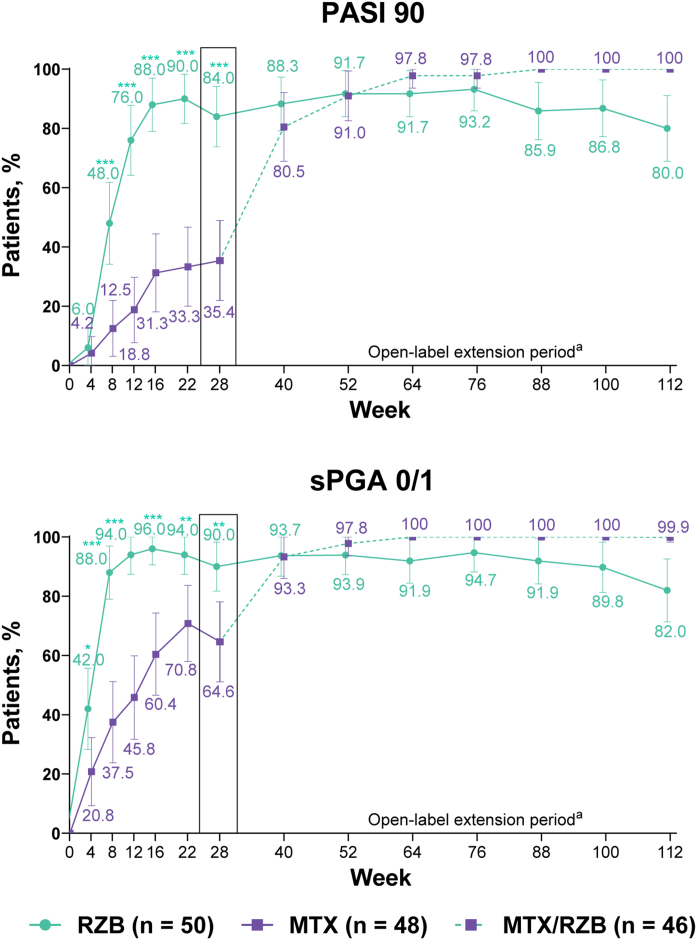
Primary clinical efficacy results. Highlighted data at week 28 denote primary efficacy endpoints. Non-responder imputation was used through week 28; non-responder imputation incorporating multiple imputation for data missing due to COVID-19 was used after week 28. MTX, Methotrexate; PASI90, ≥ 90% improvement in Psoriasis Area and Severity Index; RZB, Risankizumab; sPGA 0/1, static Physician’s Global Assessment of clear or almost clear. *p ≤ 0.05, **p ≤ 0.01, ***p < 0.001 versus MTX. ^a^ Response rates at later timepoints (after week 40) may be partially impacted by a higher frequency of missing data in the RZB group versus the MTX/RZB group.

**Figure 4:**
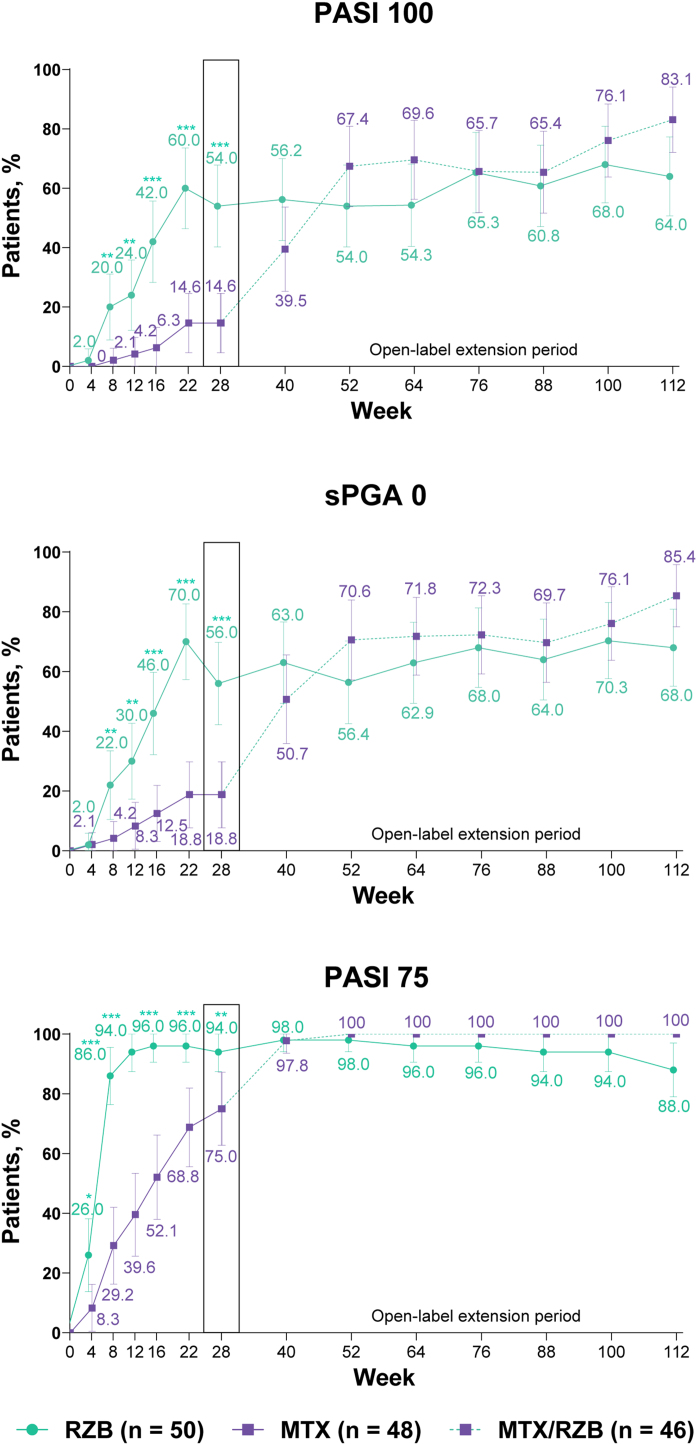
Ranked secondary clinical efficacy results. Highlighted data at week 28 denote ranked secondary efficacy endpoints. Non-responder imputation was used through week 28; non-responder imputation incorporating multiple imputation for data missing due to COVID-19 was used after week 28. MTX, methotrexate; PASI75/100, ≥ 75%/ ≥ 100% improvement in Psoriasis Area and Severity Index; RZB, risankizumab; sPGA0, static Physician’s Global Assessment of clear. *p ≤ 0.05, **p ≤ 0.01, ***p < 0.001 versus MTX.

After 28 weeks of treatment, significantly higher proportions of patients treated with risankizumab achieved the primary efficacy endpoints compared with patients treated with methotrexate (Fig. 3). PASI90 was achieved by 84.0% and 35.4% of patients in the risankizumab and methotrexate groups at week 28 (p ≤ 0.001), and sPGA 0/1 was achieved in 90.0% and 64.6% of patients in the risankizumab and methotrexate groups (p ≤ 0.001). A significantly greater proportion of patients receiving risankizumab also achieved secondary endpoints of PASI100, sPGA0, and PASI75 at week 28 compared with patients receiving methotrexate (Fig. 4).

**Figure 5:**
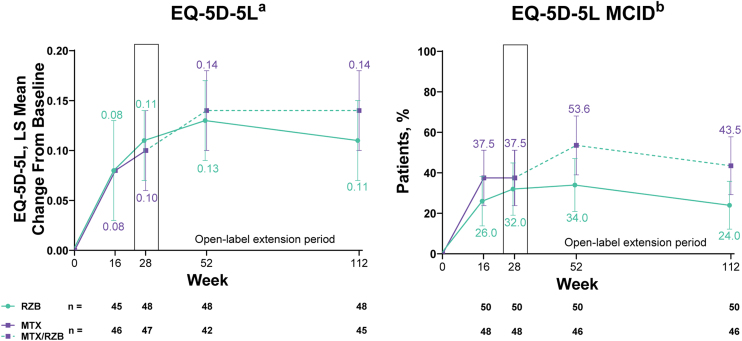
Ranked secondary quality-of-life efficacy results. Highlighted data at week 28 denote ranked secondary efficacy endpoints. MCID was defined as ≥0.1 increase in EQ-5D-5L from baseline. EQ-5D-5L, European Quality of Life 5 Dimensions; LS, least squares; MCID, Minimal Clinically Important Difference; MTX, Methotrexate; RZB, Risankizumab. ^a^ Last-observation-carried-forward imputation was used. ^b^ Non-responder imputation was used through week 28; non-responder imputation incorporating multiple imputation for data missing due to COVID-19 was used after week 28.

Improvements in patient-reported outcomes measuring health-related quality of life (HRQoL) were observed in both risankizumab and methotrexate groups. By week 28 of treatment, changes in EQ-5D-5L index scores and proportion of patients achieving the MCID in EQ-5D-5L index scores numerically increased from baseline in both groups with no significant differences in outcomes between patients treated with risankizumab versus methotrexate (Fig. 5). Among patients with baseline EQ-5D-5L index scores ≤0.9, the least squares mean change from baseline (95% CI) in EQ-5D-5L index score at week 28 was similar in both groups (0.16 [0.10, 0.23] with risankizumab and 0.17 [0.11, 0.23] with methotrexate). In the post hoc analysis evaluating patients with baseline EQ-5D-5L index scores ≤0.9, the authors observed that the proportion of patients achieving the MCID in EQ-5D-5L index scores at week 28 was numerically greater, but not significantly different, in the risankizumab group versus the methotrexate group (61.5% vs. 54.5%). At week 28, a significantly greater proportion of patients achieved DLQI 0 in the risankizumab group versus patients in the methotrexate group (Supplementary Fig. S1).

#### Long-term open-label extension analysis

PASI and sPGA responses were generally sustained through week 112 among patients who continued risankizumab treatment in the OLE period; the numbers of patients with missing data during the OLE period are shown in Supplementary Table S1. Improvements in PASI and sPGA outcomes were observed from weeks 28 to 112 among patients who were initially randomized to methotrexate in the double-blind period and then switched to risankizumab in the OLE period. The EQ-5D-5L index score response rates generally remained stable through 112 weeks of therapy, regardless of the treatment group. From weeks 28 to 112, DLQI 0 response rates remained stable among patients receiving continuous risankizumab and increased among patients who switched from methotrexate to risankizumab.

### Safety

During the double-blind period, the proportion of patients with TEAEs was slightly higher in the methotrexate treatment group (83.3%) than in the risankizumab group (77.6%; Table 2). However, TEAE profiles were generally similar between treatment groups, and the rates of serious AEs, severe AEs, and TEAEs of safety interest were generally low in both groups, with no meaningful differences between the methotrexate and risankizumab groups. Rates of serious AEs were < 5% in the risankizumab group, and none of the serious AEs in either group were deemed related to the study drug by the investigator. The most frequently reported TEAEs (≥ 5% of patients) in the risankizumab group were headache, diarrhea, arthralgia, and anxiety. The most frequently reported TEAEs (≥5% of patients) in the methotrexate group were nausea, headache, influenza, and increased alanine aminotransferase levels. No patients discontinued the study due to TEAEs in the risankizumab group. There were no cases of tuberculosis, malignancy, serious hypersensitivity, or adjudicated major adverse cardiovascular events in either group. Though numerically higher transaminase elevations compared with baseline were observed with methotrexate (alanine transaminase, 8.0 U/L; aspartate aminotransferase, 5.0 U/L) versus risankizumab (≤ 1.1 U/L change in aspartate aminotransferase or alanine transaminase), mean levels remained within the normal range for both groups.

**Table 2:** Overview of treatment-emergent adverse events (primary analysis double-blind period: weeks 0–28).

Parameter	Double-blind period	
	RZB 150 mg (n = 49)	MTX 5–25 mg (n = 48)
	n (%)	n (%)
Any TEAE	38 (77.6)	40 (83.3)
Serious TEAE	2 (4.1)	4 (8.3)
TEAE leading to discontinuation of study drug	0	2 (4.2)^a^
Most common TEAEs (≥ 5% of patients)		
Headache	9 (18.4)	5 (10.4)
Nausea	2 (4.1)	9 (18.8)
Influenza	2 (4.1)	5 (10.4)
Diarrhea	5 (10.2)	3 (6.3)
Nasopharyngitis	2 (4.1)	4 (8.3)
Alanine aminotransferase increased	2 (4.1)	4 (8.3)
Abdominal pain	0	4 (8.3)
Arthralgia	3 (6.1)	1 (2.1)
Back pain	3 (6.1)	2 (4.2)
Dizziness	3 (6.1)	0
Anxiety	3 (6.1)	0
Skin exfoliation	3 (6.1)	0
Pyrexia	0	4 (8.3)
TEAEs of safety interest		
Adjudicated MACE	0	0
Adjudicated anaphylaxis	0	0
Serious infections	0	1 (2.1)
Opportunistic infections	0	0
Active TB	0	0
Malignant tumors	0	0
Serious hypersensitivity	0	0
Hepatic events	4 (8.2)	5 (10.4)
Deaths	0	1 (2.1)^b^

MACE, Major Adverse Cardiovascular Events; MTX, Methotrexate; RZB, Risankizumab; TB, Tuberculosis; TEAE, Treatment-Emergent Adverse Events.

a Two patients discontinued study treatment due to TEAEs assessed by the investigator as possibly related to study drug (one due to abdominal pain and appendicitis and one due to urinary tract infection, abdominal pain, and vomiting).

b One death in the MTX group was due to serious infections of appendicitis, peritonitis, and abdominal sepsis, none of which were considered possibly related to the study drug.

In the long-term extension period, TEAE rates remained similar among patients who continued with open-label risankizumab or switched from methotrexate to open-label risankizumab (Table 3). Incidence of serious AEs, TEAEs leading to discontinuation, and TEAEs of safety interest remained low. After patients switched from methotrexate to risankizumab, the safety profile of risankizumab remained consistent, and TEAE rates were stable with long-term risankizumab treatment. Rates of TEAEs of safety interest were generally low in both groups, and there were no serious hypersensitivity reactions. In the group receiving continuous risankizumab, one adjudicated major adverse cardiovascular event in a patient with multiple cardiovascular risk factors was reported. All fungal infections were related to skin or vaginal *Candida*. Among patients who received continuous risankizumab, pancreatic cancer was reported for one patient who was a former smoker. Overall, the mean changes from baseline in laboratory parameters were generally small among patients who switched from methotrexate to risankizumab and patients who continued risankizumab treatment; laboratory values remained within normal ranges throughout the OLE period.

**Table 3:** Overview of treatment-emergent adverse events (long-term analysis: weeks 28–112).

Events	Open-label extension period^a^		
	RZB/RZB	MTX/RZB	All RZB
	n = 49	n = 46	n = 95
	PYs = 83.4	PYs = 81.1	PYs = 190.9
	E/100 PYs	E/100 PYs	E/100 PYs
Any TEAE	144 (172.7)	172 (212.1)	451 (236.2)
Serious TEAE	3 (3.6)	9 (11.1)	17 (8.9)
TEAE leading to discontinuation of study drug	2 (2.4)	0	2 (1.0)
Most common TEAEs (≥ 5 E/100 PY)			
Headache	4 (4.8)	7 (8.6)	23 (12.0)
COVID-19	3 (3.6)	9 (11.1)	12 (6.3)
Hypertension	3 (3.6)	8 (9.9)	12 (6.3)
Anxiety	5 (6.0)	4 (4.9)	12 (6.3)
Nasopharyngitis	7 (8.4)	2 (2.5)	11 (5.8)
Blood creatine phosphokinase increased	5 (6.0)	4 (4.9)	9 (4.7)
Upper respiratory tract infection	1 (1.2)	7 (8.6)	10 (5.2)
Pain in extremity	0	6 (7.4)	8 (4.2)
Nephrolithiasis	5 (6.0)	1 (1.2)	7 (3.7)
Hypertensive crisis	0	5 (6.2)	6 (3.1)
Rhinitis	5 (6.0)	0	5 (2.6)
TEAEs of safety interest			
Adjudicated MACE	1 (1.2)^b^	0	1 (0.5)^b^
Serious infections	0	6 (7.4)	6 (3.1)
Opportunistic infections excluding TB and herpes zoster	0	0	0
Active TB	0	0	0
Malignant tumors excluding NMSC	1 (1.2)	0	1 (0.5)
NMSC	0	1 (1.2)	1 (0.5)
Hepatic events	5 (6.0)	3 (3.7)	12 (6.3)
Serious hypersensitivity	0	0	0
Deaths	0	0	0

E, Events; MACE, Major Adverse Cardiovascular Events; MTX, Methotrexate; NMSC, Non-Melanoma Skin Cancer; PYs, Patient-Years; RZB, Risankizumab; TB, Tuberculosis; TEAE, Treatment-Emergent Adverse Events.

a Safety includes data from weeks 28–112 (open-label extension period), including those patients who started on RZB 150 mg or MTX 5–25 mg at randomization and continued with or switched to open-label RZB 150 mg after week 28.

b A serious myocardial infarction event occurred in a 55-year-old male with a medical history of arterial hypertension, diabetes, hyperlipidemia, obesity, and a history of smoking; the event was assessed as having no reasonable possibility of a relationship to the study drug.

## Discussion

In this phase 3 study, risankizumab demonstrated superiority over methotrexate in the proportion of patients achieving PASI90 and sPGA0/1. The response rates for PASI90 and achievement of sPGA0/1 were consistent with those reported for the pivotal global studies in psoriasis for risankizumab.^10^ Further, significant improvements were shown with risankizumab treatment in the ranked secondary efficacy endpoints, including total skin clearance (sPGA0 and PASI100). Efficacy was maintained through week 112 for patients who initially received risankizumab, and outcomes improved through week 112 for patients who started on methotrexate and then switched to risankizumab at week 28.

Improvements in HRQoL are important in assessing the overall benefits of new therapies. In this study, EQ-5D-5L index score changes and MCID response rates were not different between the risankizumab and methotrexate groups. Notably, EQ-5D-5L is a short, generic, non-disease-specific measure of health status and can be less sensitive to improvements in HRQoL experienced by people with psoriasis when their symptoms improve. Nearly twice as many patients treated with risankizumab versus methotrexate achieved the exploratory endpoint of DLQI 0 at week 28. DLQI0 responses were maintained through 112 weeks of therapy for patients who continued risankizumab treatment, and outcomes improved through week 112 for patients who switched from methotrexate to risankizumab at week 28. Results from previous studies have also shown that patients treated with risankizumab experience significant and clinically meaningful improvements in their HRQoL when assessed by more comprehensive and/or disease-specific HRQoL instruments and patient-reported outcomes (e.g., tools like the DLQI, Psoriasis Symptom Scale, and 36-Item Short Form Health Survey).^9^, ^10^, ^11^, ^12^, ^15^

Risankizumab was well tolerated, and no new safety concerns were identified. The TEAEs of anxiety and dizziness (reported for three patients each in the risankizumab arm during the double-blind period) were not identified as common adverse events in an integrated analysis of the safety of risankizumab in patients with psoriasis across 17 global clinical trials.^16^ The safety profile of risankizumab observed through 112 weeks of therapy was consistent with the safety profile of long-term risankizumab treatment in patients with psoriasis.

A limitation of this study is the small sample size due to the difficulty of recruiting patients with moderate-to-severe psoriasis who have not previously used methotrexate.

Methotrexate has been widely used as a first-line treatment for psoriasis in Brazil. The most recently published Brazilian guideline for moderate-to-severe psoriasis (2019) suggests that biologics should be reserved for patients with no response to treatment, contraindications for using a specific drug, or intolerance to at least one systemic drug or phototherapy.^17^ Physicians’ reluctance to choose a biologic as first-line therapy may be connected to access restrictions associated with cost or partly due to the lack of data from clinical studies that compare traditional methotrexate therapy with biologics. The present results support that risankizumab provides greater efficacy than methotrexate in patients with moderate-to-severe plaque psoriasis, with no additional safety risks.

## Conclusions

Risankizumab demonstrated significant improvements in psoriasis symptoms and superior efficacy compared with methotrexate at week 28. Further, the effectiveness of risankizumab was maintained over time through week 112, demonstrating that durability and improvements in efficacy were demonstrated in patients who switched from methotrexate to risankizumab in the long-term OLE period. The safety profile of risankizumab was consistent with the safety profile reported in the other risankizumab studies in psoriasis. Overall, these results confirm risankizumab as a viable treatment option in patients with moderate-to-severe plaque psoriasis.

## Data availability statement

AbbVie is committed to responsible data sharing regarding the clinical trials the authors sponsor. This includes access to anonymized individual and trial-level data (analysis data sets), as well as other information (e.g., protocols, clinical study reports, or analysis plans), as long as the trials are not part of an ongoing or planned regulatory submission. This includes requests for clinical trial data for unlicensed products and indications.

These clinical trial data can be requested by any qualified researchers who engage in rigorous, independent, scientific research, and will be provided following review and approval of a research proposal, statistical analysis plan, and execution of a data sharing agreement. Data requests can be submitted at any time after approval in the US and Europe and after acceptance of this manuscript for publication. The data will be accessible for 12 months, with possible extensions considered. For more information on the process or to submit a request, visit the following link: https://vivli.org/ourmember/abbvie/, then select “Home”.

## Financial support

AbbVie funded this study and participated in the study design, research, analysis, data collection, interpretation of data, reviewing, and approval of the publication. All authors had access to relevant data and participated in the drafting, review, and approval of this publication. No honoraria or payments were made for authorship. Medical writing support was provided by Melissa Julyanti, PharmD, of JB Ashtin, and funded by AbbVie.

## Author’s contribution

Tania F. Cestari: Approval of the final version of the manuscript; data collection, analysis, and interpretation; manuscript critical review; preparation and writing of the manuscript.

Cacilda da Silva Souza: Approval of the final version of the manuscript; data collection, analysis, and interpretation; manuscript critical review; preparation and writing of the manuscript.

Luna Azulay-Abulafia: Approval of the final version of the manuscript; data collection, analysis, and interpretation; manuscript critical review; preparation and writing of the manuscript.

Ricardo Romiti: Approval of the final version of the manuscript; data collection, analysis, and interpretation; manuscript critical review; preparation and writing of the manuscript.

André V.E. Carvalho: Approval of the final version of the manuscript; data collection, analysis, and interpretation; manuscript critical review; preparation and writing of the manuscript.

Caio César Silva de Castro: Approval of the final version of the manuscript; data collection, analysis, and interpretation; manuscript critical review; preparation and writing of the manuscript.

Silvio Alencar Marques: Approval of the final version of the manuscript; data collection, analysis, and interpretation; manuscript critical review; preparation and writing of the manuscript.

João Roberto Antonio: Approval of the final version of the manuscript; data collection, analysis, and interpretation; manuscript critical review; preparation and writing of the manuscript.

Lincoln Fabrício: Approval of the final version of the manuscript; data collection, analysis, and interpretation; manuscript critical review; preparation and writing of the manuscript.

Ahmed M. Soliman: Approval of the final version of the manuscript; data collection, analysis, and interpretation; manuscript critical review; preparation and writing of the manuscript.

Tianshuang Wu: Approval of the final version of the manuscript; data collection, analysis, and interpretation; manuscript critical review; preparation and writing of the manuscript; statistical analysis; study conception and planning.

Ranjeeta Sinvhal: Approval of the final version of the manuscript; data collection, analysis, and interpretation; manuscript critical review; preparation and writing of the manuscript; study conception and planning.

Vassilis Stakias: Approval of the final version of the manuscript; data collection, analysis, and interpretation; manuscript critical review; preparation and writing of the manuscript.

Alexandra P. Song: Approval of the final version of the manuscript; data collection, analysis, and interpretation; manuscript critical review; preparation and writing of the manuscript.

Jasmina Kalabic: Approval of the final version of the manuscript; data collection, analysis, and interpretation; manuscript critical review; preparation and writing of the manuscript.

Naomi Martin: Approval of the final version of the manuscript; data collection, analysis, and interpretation; manuscript critical review; preparation and writing of the manuscript.

Luiza Keiko Matsuka Oyafuso: approval of the final version of the manuscript; data collection, analysis, and interpretation; manuscript critical review; preparation and writing of the manuscript.

## Conflicts of interest

Dr. Cestari has served as a speaker, consultant, and/or investigator for AbbVie, Janssen‐Cilag, La Roche‐Posay, LEO Pharma, Novartis, Pierre‐Fabre, and Vichy.

Dr. da Silva Souza has served as a consultant and/or investigator for AbbVie, Boehringer Ingelheim, Janssen‐Cilag, LEO Pharma, and Novartis.

Dr. Azulay-Abulafia has served as a consultant and/or investigator for AbbVie, Janssen‐Cilag, LEO Pharma, Lilly, Novartis, and Pfizer.

Dr. Romiti has served as a consultant, speaker, and/or investigator for AbbVie, Bioderma, Boehringer Ingelheim, Galderma, Janssen‐Cilag, LEO Pharma, Lilly, Novartis, Pfizer, and UCB.

Dr. Carvalho has served as a consultant, speaker, and/or investigator for AbbVie, AMGEN, Boehringer Ingelheim, GSK, Janssen‐Cilag, LEO Pharma, Lilly, and Novartis.

Dr. de Castro has served as a consultant, speaker, and/or investigator for AbbVie, Janssen, Novartis, and Pfizer.

Dr. Marques has served as a local investigator and received grants/research funding from Janssen.

Dr. Antonio has served as a speaker and principal investigator for AbbVie, Janssen, and Novartis.

Dr. Fabrício has served as a speaker for Abbott, AbbVie, Bayer, Bioderma, Biolab, Boticário, Galderma, Hypermarcas, Isdin, Janssen, La Roche‐Posay, LEO Pharma, Pfizer, and Stiefel/GSK, and has received sponsorship for scientific events from Abbott, AbbVie, Bayer, Bioderma, Galderma, Isdin, Janssen, La Roche‐Posay, LEO Pharma, MSD, Novartis, and Pfizer. He has participated in advisory boards for Bayer, Janssen, La Roche‐Posay, LEO Pharma, and MSD.

Dr. Soliman, Dr. Wu, Dr. Sinvhal, Dr. Stakias, Dr. Song, and Dr. Kalabic are employees of AbbVie and may hold AbbVie stock, stock options, and/or patents.

Dr. Martin is a former employee of AbbVie.

Dr. Oyafuso has served as a consultant and/or investigator for AbbVie, Janssen‐Cilag, and Novartis.
