# Supplementary material for: Efficacy and safety of risankizumab versus methotrexate in patients with moderate-to-severe plaque psoriasis: results from IMMbrace, a randomized, double-blind, phase 3 study with an open-label extension period in Brazil
Source: An Bras Dermatol. 2024 Dec 7;100(2):260–71. doi: 10.1016/j.abd.2024.08.002 (PMC11962814; doi:10.1016/j.abd.2024.08.002)
Supplement: Supplementary file 1 [file mmc1.pdf]

## Appendix A. Supplementary data

**Efficacy and safety of risankizumab versus methotrexate in patients with moderate-to-severe plaque psoriasis: results from IMMbrace, a randomized, double-blind, phase 3 study with an open-label extension period in Brazil**

**Supplementary Table S1** Patients with missing data for PASI 90 and sPGA 0/1 over the open-label extension period.

**Supplementary Figure S1** Proportion of patients achieving DLQI 0.

**Appendix A. Supplementary data**

**Table S1** Patients with missing data for PASI 90 and sPGA 0/1 over the open-label extension period.

| | Week 40 | | Week 52 | | Week 64 | | Week 76 | | Week 88 | | Week 100 | | Week 112 | |
|---|---|---|---|---|---|---|---|---|---|---|---|---|---|---|
| | RZB n = 50 | MTX/ RZB n = 46 | RZB n = 50 | MTX/ RZB n = 46 | RZB n = 50 | MTX/ RZB n = 46 | RZB n = 50 | MTX/ RZB n = 46 | RZB n = 50 | MTX/ RZB n = 46 | RZB n = 50 | MTX/ RZB n = 46 | RZB n = 50 | MTX/ RZB n = 46 |
| **PASI 90, n (%)** | | | | | | | | | | | | | | |
| Reason for missing data | | | | | | | | | | | | | | |
| COVID-19 | 1 (2.0) | 3 (6.5) | 1 (2.0) | 2 (4.3) | 1 (2.0) | 1 (2.2) | 4 (8.0) | 0 | 3 (6.0) | 0 | 1 (2.0) | 0 | 0 | 1 (2.2) |
| Other | 1 (2.0) | 1 (2.2) | 1 (2.0) | 0 | 2 (4.0) | 0 | 2 (4.0) | 0 | 3 (6.0) | 0 | 3 (6.0) | 0 | 6 (12.0) | 0 |
| **sPGA 0/1, n (%)** | | | | | | | | | | | | | | |
| Reason for missing data | | | | | | | | | | | | | | |
| COVID-19 | 1 (2.0) | 3 (6.5) | 1 (2.0) | 1 (2.2) | 1 (2.0) | 1 (2.2) | 4 (8.0) | 0 | 3 (6.0) | 0 | 1 (2.0) | 0 | 0 | 1 (2.2) |
| Other | 1 (2.0) | 1 (2.2) | 1 (2.0) | 0 | 2 (4.0) | 0 | 2 (4.0) | 0 | 3 (6.0) | 0 | 3 (6.0) | 0 | 6 (12.0) | 0 |

MTX, methotrexate; PASI 90; ≥90% improvement in Psoriasis Area and Severity Index; RZB, risankizumab; sPGA 0/1, static Physician's Global Assessment of clear or almost clear.

**Figure S1** Proportion of patients achieving DLQI 0.

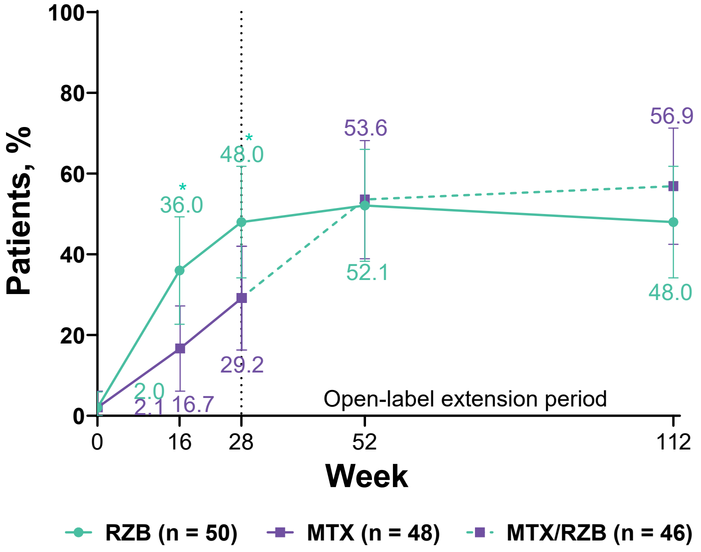


Non-responder imputation was used through week 28; non-responder imputation incorporating multiple imputation for data missing due to COVID-19 was used after week 28.
DLQI 0, Dermatology Life Quality Index score of no effect on patient's life; MTX, methotrexate; RZB, risankizumab.
* p ≤0.05 versus MTX.
